# Extremely-Low-Cycle Fatigue Damage for Beam-to-Column Welded Joints Using Structural Details

**DOI:** 10.3390/ma13071768

**Published:** 2020-04-09

**Authors:** Lizhen Huang, Weilian Qu, Ernian Zhao

**Affiliations:** 1 School of Civil Engineering, Hubei Engineering University, Xiaogan 432000, China; 2 Hubei Key Laboratory of Roadway Bridge & Structure Engineering, Wuhan University of Technology, Wuhan 430070, China; 3 School of Civil Engineering, Shandong Jianzhu University, Jinan 250101, China

**Keywords:** constructional engineering, welded joint, structural details, multiaxial low-cycle fatigue, fatigue life prediction

## Abstract

The multiaxial fatigue critical plane method can be used to evaluate the extremely-low-cycle fatigue (ELCF) damage of beam-to-column welded joints in steel frameworks subjected to strong seismic activity. In this paper, fatigue damage models using structural detail parameters are studied. Firstly, the fatigue properties obtained from experiments are adopted to assess ELCF life for steel frameworks. In these experiments, two types of welded specimens, namely, plate butt weld (PB) and cruciform load-carrying groove weld (CLG), are designed according to the structural details of steel beam and box column joints, in which both structural details and welded factors are taken into account. Secondly, experiments are performed on three full-scale steel welded beam-to-column joints to determine the contribution of stress and/or strain to damage parameters. Finally, we introduce a modification of the most popular fatigue damage model of Fatemi and Socie (FS), modified by us in a previous study, for damage evaluation, and compare this with Shang and Wang (SW) in order to examine the applicability of the fatigue properties of PB and CLG. This study shows that the modified FS model using the fatigue properties of CLG can predict the crack initiation life and evaluate the damage of beam-to-column welded joints, and can be subsequently used for further investigation of the damage evolution law.

## 1. Introduction

Steel beam-to-column welded joints may undergo extremely-low-cycle fatigue (ELCF) during strong seismic activity [1,2,3], which is usually characterized by a few reverse loading cycles (in general, less than 20) with large strain amplitudes before failure. Welded joints are crucial components in antiseismic steel structures; for instance, the 1994 Northridge and 1995 Kobe earthquakes which caused severe damage to steel structures, where cracks were initiated in welded joints and eventually caused the collapse of whole structures [4,5]. The structural details of welding defects present possible new critical positions for ELCF crack initiation, which plays an important role in assessing fatigue crack initiation life and damage. The numerous available methods for seismic damage evaluation of steel frame structures are classified into two categories. The first category mainly focuses on establishing the damage evaluation model using macroparameters, such as plastic deformation [6], displacement amplitude [7,8], and rotation amplitude [9] combined with the low-cycle S-N curve, which describe the relationship between strain and the number of cycles to failure. The second category is based on cumulative damage of low-cycle fatigue, which takes plastic deformation of structures or components [10,11,12], energy dissipation [13,14,15,16,17], or the combination of these two variables [18,19,20,21,22] as a seismic damage parameter. In addition, numerous experiments have also been carried out to evaluate the structural damage of seismic excitations. However, existing studies mostly focus on the ductility and energy dissipation capability of the whole structure or structural component, and ignore the local details of welded joints and the fatigue crack initiation mechanics during the ELCF fracture process, which may lead to inaccurate damage evaluation results.

With the application of the micromechanical approach in fracture predictions of welded joints, researchers have paid attention to investigating the fatigue failure behavior [23,24,25,26,27] and severe seismic damage assessments [28,29,30] from the aspect of structural details. From these aspects, the results can be more suitable than traditional methods [26,28]. However, welded joints are always in multiaxial and nonproportional stress states due to their geometrically discontinuous properties, which result in more fatigue damage [31]. Therefore, a suitable model is necessary to correctly describe the effect of multiaxial and nonproportional stress states in fracture predictions for welded joints. The widely accepted critical plane approach is used to determine the damage parameter in a multiaxial nonproportional fatigue damage model. The critical plane approach usually uses the maximum shear plane as the critical damage plane, and takes the maximum shear strain $\gamma_{\max}$ and normal strain $\varepsilon_{n}$ on the critical plane as the two basic damage parameters. The most popular multiaxial fatigue damage model combining strain with stress components is that proposed by Fatemi and Socie (FS) [32], which considers the nonproportional influence. Based on this study, numerous researchers have improved the nonproportionality parameter in the FS model based on their own research backgrounds [31,33,34,35], resulting in specific engineering applications. Another popular damage parameter using a path-independent parameter, $\Delta \varepsilon_{eq}^{cr}/2$, composed of normal strain excursion $\varepsilon_{n}^{*}$ and maximum shear strain $\gamma_{\max}$, was proposed by Shang and Wang (SW) [36], but has not yet been used in steel welded joints. Furthermore, the application of multiaxial fatigue damage modeling in ELCF failure prediction for steel welded joints is also limited. In conclusion, the research and application of multiaxial fatigue damage evaluation of steel beam-to-column welded joints under ELCF loading conditions still require further study.

This paper aims to propose a fatigue damage parameter for ELCF crack initiation life prediction and damage assessment for steel beam-to-column welded joints, in which the I-beam and box-column profile is considered in the joint. Firstly, ELCF tests are carried out by designing two types of welded specimens according to welded structural details for obtaining material parameters in multiaxial fatigue damage modeling. Secondly, reverse cyclic loading tests are performed on three full-scale welded joints to obtain the local stress and strain response of welded joints, and the experimental data are used to form the fatigue damage parameter. In addition, the FS model is modified from our previous study using the nonproportionality factor $k^{*}$, based on both the nonproportionality of strain path Φ and material nonproportional hardening coefficient L. Finally, the modified FS model and the SW model are checked for their accuracy and reliability in assessing the fatigue damage of steel beam-to-column welded joints.

## 2. Experiments

In this section, ELCF tests of welded structural details and reverse cyclic loading tests of I-beam to box-column welded joints are carried out to obtain the parameters in the multiaxial fatigue damage model.

### 2.1. ELCF Test

The material and fatigue parameters in the heat affected zone (HAZ) are usually much lower and worse than the corresponding properties of the base materials. Indeed, fatigue failure of welded steel structures may happen at the HAZ. However, according to the damage investigation of the Northridge earthquake in 1994, it was found that about 100 steel frameworks suffered from different degrees of damage at the beam-to-column joints. It was also shown that the cracks originating in the weld zone occupied comprised as much as 90% of the total number, while only about 10% originated from the base metal. There were obvious cracks initiating at the weld seam of the beam-to-column welded joints, and the cracks propagated to the members connected to the joints, causing the failure of the steel frame. It is difficult to determine the location of the HAZ and to take a sample for analysis. Therefore, we focus on the weld zone in this paper.

**Figure 1:**
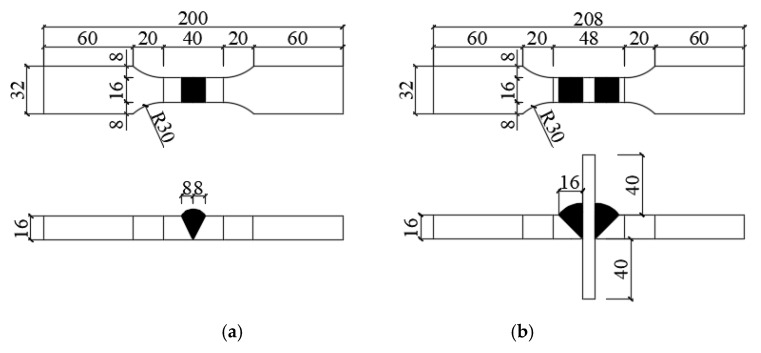
Design of welded specimens (mm): (**a**) plate butt (PB); (**b**) cruciform load-carrying groove (CLG).

As shown in Figure 1, two types of welded structural detail specimens were designed to carry out ELCF tests in order to investigate the fatigue properties of the welded structural details of box-column joints in a high-rise steel frame structure. There were twelve specimens in each type. The first type, namely, plate butt (PB) weld specimens, were used to simulate the welded splicing between the beam plate or between the column plate. The second type, namely, cruciform load-carrying groove (CLG) weld specimens, were used to simulate the welded connection between the beam flange plate and column flange plate. Both of the weld forms were single-sided groove welds. The base material was Q235 low-carbon steel. The mechanical properties of tested material are listed in Table 1.

**Figure 2:**
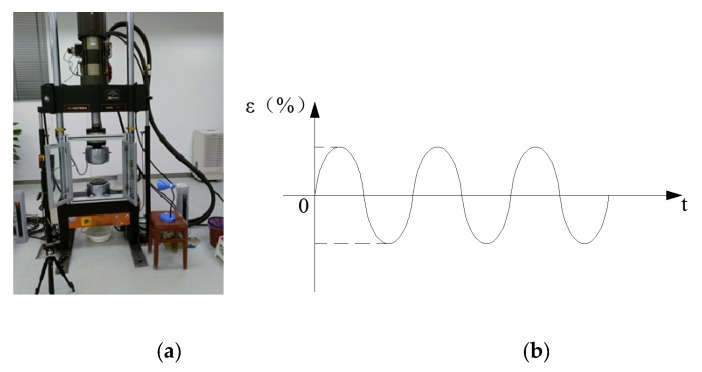
(**a**)Test equipment; (**b**) loading history.

These fatigue tests were conducted on an INSTRON1342 fatigue test machine, and controlled by uniaxial constant strain amplitude using sinusoidal wave forms, as shown in Figure 2. The strain ratio, i.e., the ratio of maximum strain to minimum strain, was −1 in these tests.

**Figure 3:**
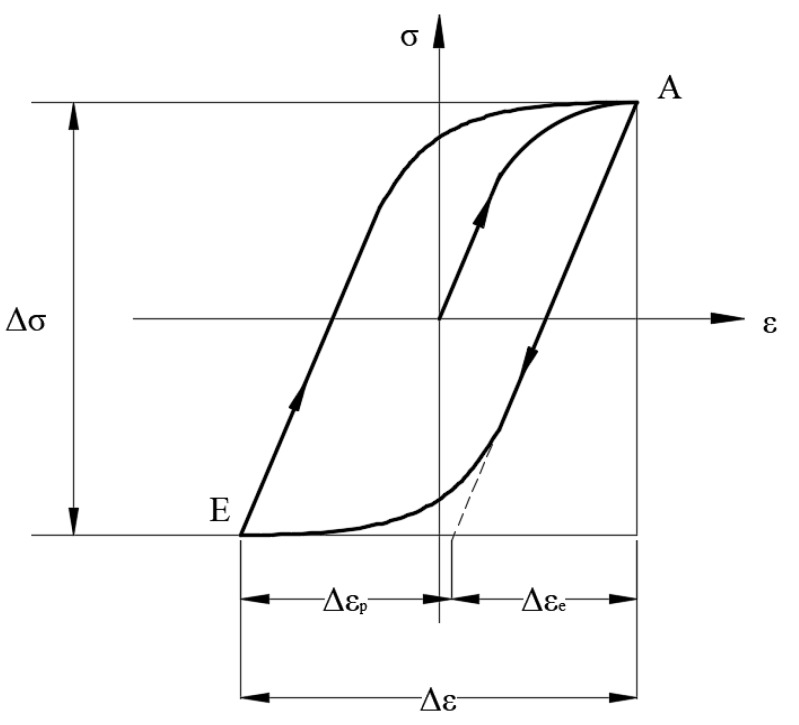
Cyclic stress–strain hysteresis curve.

The experimental results were listed in [27] as shown in Table 2. The identifier for the specimen number corresponds to the loading condition; for instance, PB04 and PB10 mean that the tests for PB specimens were controlled by total strain amplitudes of 0.004 and 0.010, respectively. The meanings of the symbols in Table 2 are the same as those in Equations (1) and (2). For either loading conditions, $\frac{\Delta \varepsilon }{2}$, $\frac{\Delta \varepsilon_{e}}{2}$, $\frac{\Delta \varepsilon_{p}}{2}$ and $\frac{\Delta \sigma }{2}$ are obtained based on the stable hysteresis loop curve, as shown in Figure 3.

The fatigue properties of the welded structural details, which are correlated by the Manson–Coffin equation expressed by Equation (1), are listed in Table 3. Also listed in this table are the cyclic stress–strain properties when the stable hysteresis loop curve is fitted into the Ramberg–Osgood relation expressed by Equation (2). The fatigue and cyclic stress–strain properties are fitted using General model Power1 in MATLAB, that is, $f\left(x\right)=a\cdot x^{b}$.

(1) $$\frac{\Delta \varepsilon }{2}=\frac{\Delta \varepsilon_{e}}{2}+\frac{\Delta \varepsilon_{p}}{2}=\frac{{\sigma^{\prime }}_{f}}{E}{\left(2N_{f}\right)}^{b}+{\varepsilon^{\prime }}_{f}{\left(2N_{f}\right)}^{c}$$

(2) $$\frac{\Delta \varepsilon }{2}=\frac{\Delta \varepsilon_{e}}{2}+\frac{\Delta \varepsilon_{p}}{2}=\frac{\Delta \sigma }{2E}+{\left(\frac{\Delta \sigma }{2K^{\prime }}\right)}^{\frac{1}{n^{\prime }}}$$

where E is Young’s modulus; $N_{f}$ is the number of cycles to failure, ${\sigma^{\prime }}_{f}$, ${\varepsilon^{\prime }}_{f}$ are fatigue strength coefficient and fatigue ductility coefficient, respectively, b and c are fatigue strength exponent and fatigue ductility exponent, respectively, and $\frac{\Delta \varepsilon_{e}}{2}=\frac{{\sigma^{\prime }}_{f}}{E}{\left(2N_{f}\right)}^{b}$, $\frac{\Delta \varepsilon_{p}}{2}={\varepsilon^{\prime }}_{f}{\left(2N_{f}\right)}^{c}$, and $\frac{\Delta \varepsilon }{2}=\frac{\Delta \varepsilon_{e}}{2}+\frac{\Delta \varepsilon_{p}}{2}$ are, respectively, elastic strain amplitude, plastic strain amplitude, and total strain amplitude. In Equation (2), $\frac{\Delta \sigma }{2}$ is stress amplitude, $K^{\prime }$ is the cyclic strength coefficient, and $n^{\prime }$ is the cyclic strain hardening exponent.

### 2.2. Reverse Cyclic Loading Test

**Figure 4:**
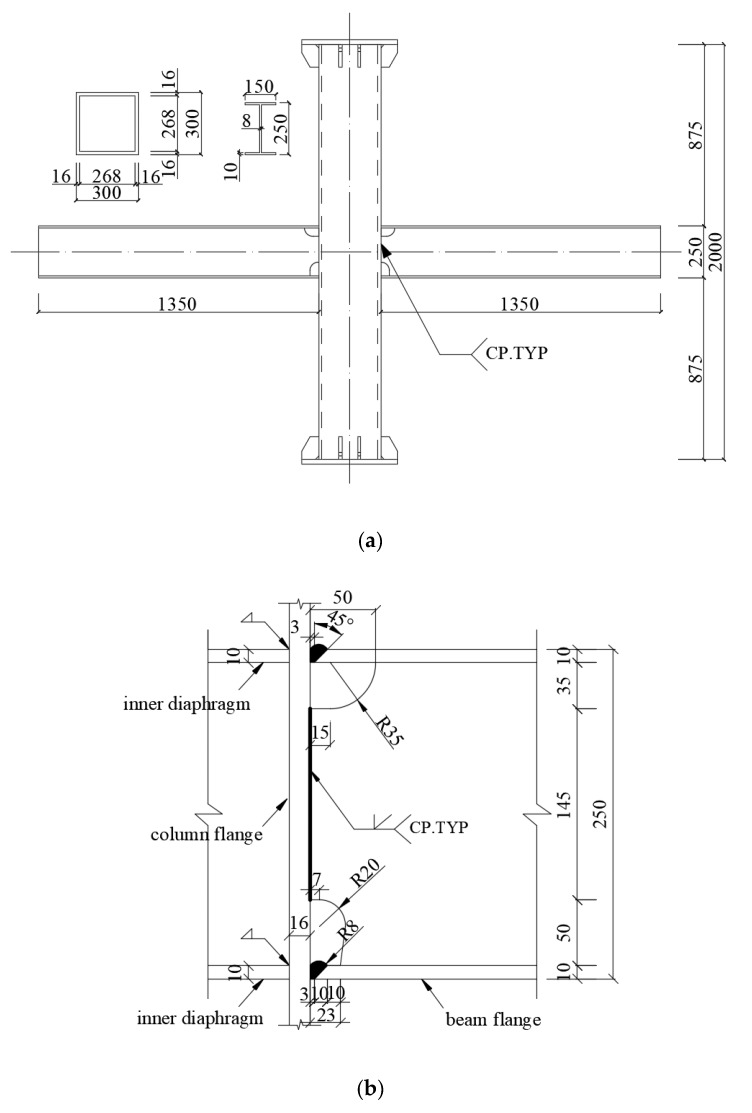
Design of specimen (mm): (**a**) beam-to-column welded joint; (**b**) welded structural details.

Three cruciform beam-to-column welded joints were designed in this experiment, in which the inflection point of the beam-to-column connection was selected. The column had box sections with a size of 300 × 300 × 16 × 16 (mm) and had a height of 2 m. The beam had an I-shaped cross-section with a size of 250 × 150 × 8 × 10 (mm) and a beam length of 1.35 m. The weld form between the beam flange and the column flange was a full penetration groove using ER50-6 gas shielded welding wire. Welding process quality inspection was performed using a digital ultrasonic flaw detector; the grade of the weld seam was I. The beam-to-column welded joint specimens and welded structural details are shown in Figure 4.

**Figure 5:**
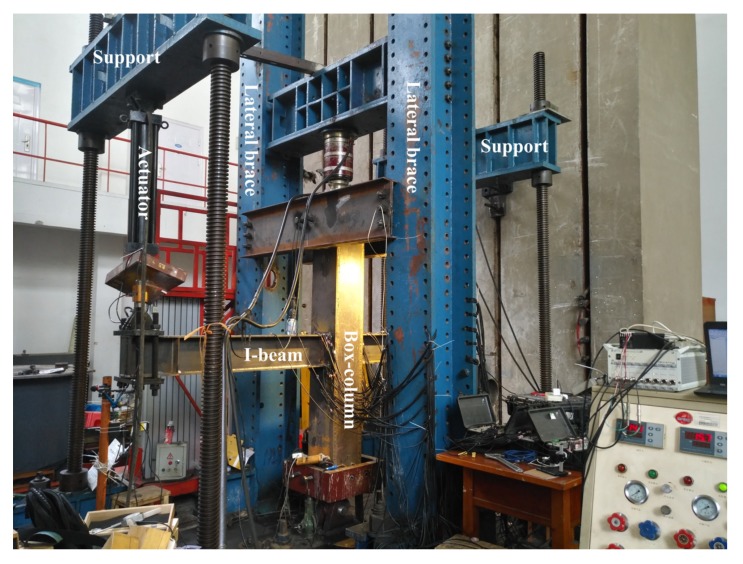
Test setup configuration.

The test device is shown in Figure 5. The DH5956 dynamic signal analysis acquisition system and multiple JM3812 multifunctional static strain data acquisition systems were applied in this test, and a 200-fold ultra-high-definition electron microscope was used to observe the cracks in the beam-to-column welded joints. To facilitate the statistics of crack growth and strain response, the left and right directions of Figure 5 are marked as west and east, respectively, and the other two orthogonal directions are marked as north and south, respectively.

**Figure 6:**
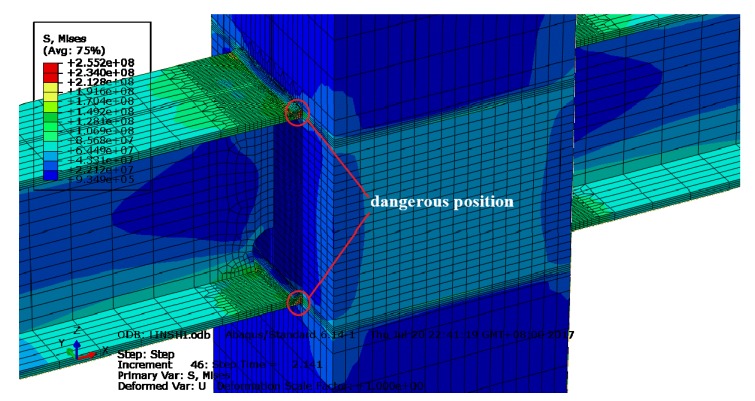
von Mises stress distribution nephogram.

**Figure 7:**
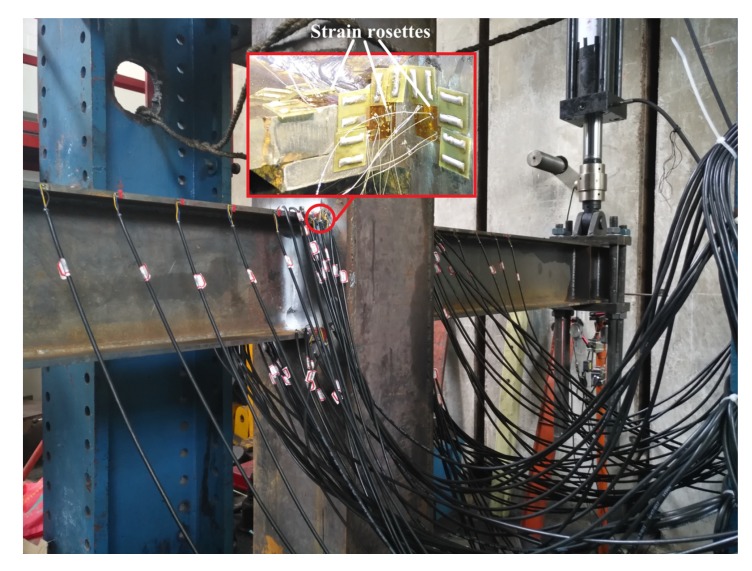
The location of strain rosettes in the specimen.

According to the numerical simulation results of ABAQUS finite element analysis, the cruciform I-beam to box-column welded joint is in yield state when the displacement is greater than 12 mm, which is the noted yield displacement. It can be seen from Figure 6 that at time moment t = 2.141s, when the simulation is controlled by yield displacement, the model has maximum von Mises stress at the weld roots of the upper and bottom flanges with a stress value of 255.2 MPa, which is greater than the yield strength value shown in Table 1. This indicates that the weld zone is in the stage of plastic deformation and the weld root on the beam flange plate of the welded connection is in a position of potential fatigue failure. Hence, strain rosettes are arranged in the vicinity of the dangerous position to obtain the strain response in this test, as shown in Figure 7. Moreover, a reciprocating displacement greater than the yield displacement is selected for the cyclic loading test in order to simulate the stress state of welded joints of the steel framework when undergoing strong earthquake actions. In the test, actuators are controlled synchronously and in reverse on the beam ends. The loading histories are shown in Table 4.

**Figure 8:**
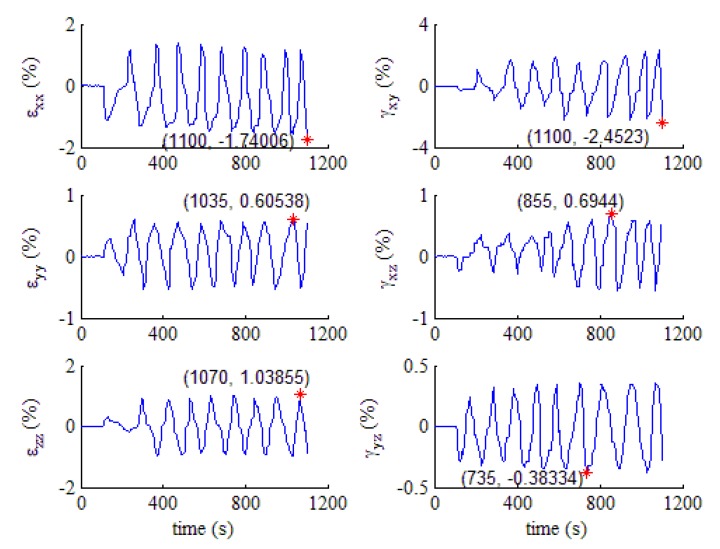
Six strain responses of NWB_2 weld. (* in the figure means the peak or valley of the strain).

The bottom flange at the northwest of the second specimen, which is marked with NWB_2, was selected to analyze the strain response. This was validated by a test showing that the weld root on the beam flange plate of the welded connection is in a position where fatigue cracks are likely to initiate. From the experiment, it was observed that under cyclic loading with a constant displacement amplitude, the tiny visible crack in NWB_2 was initiated just after the 7th cycle, i.e., at the time period between the 7th and 8th cycle. Strain data are obtained by placing three strain rosettes at the three coordinate planes in the vicinity of the weld root. Three normal strains and three shear strains of the weld root are then calculated using the elastic mechanics theory. The extracted strain response time history curves of NWB_2, ranging from the beginning of the trial to crack initiation, are drawn in Figure 8.

It can be seen from Figure 8 that the sizes of the six strain components do not change in a fixed proportion, nor do they reach the peak and/or valley at the same time. There is a certain phase difference between strains, that is, the strain response has nonproportional characteristics. Furthermore, the force characteristic is usually multiaxial in the welded zone due to complex geometric characteristics. In fact, a complex component, which has a sudden change of geometric shape, such as a notch or a weld, is always in a multiaxial stress–strain state, even though it is subjected to uniaxial load. That is, the failure of a beam-to-column welded joint which undergoes severe seismic excitation is multiaxial and nonproportional ELCF. Therefore, the widely used multiaxial fatigue critical plane method was adopted to evaluate the fatigue damage of the beam-to-column weld joint in this paper.

## 3. Methods of Evaluation

In this section, two fatigue critical plane methods are used. We outline damage analysis steps and fatigue damage parameters ranging from strain response on the critical plane to nonproportional factors, and cumulative damage criteria are introduced.

### 3.1. Damage Analysis Steps

**Figure 9:**
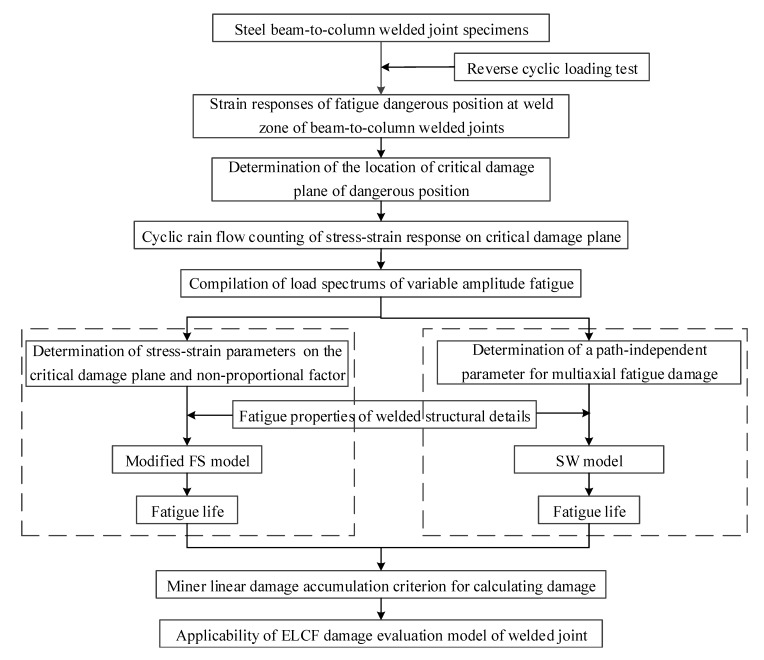
Extremely-low-cycle fatigue (ELCF) damage analysis steps.

The multiaxial fatigue analysis method is used to analyze the extremely-low-cycle fatigue damage of beam-to-column welded joints; the steps are shown in Figure 9 and summarized as follows:

(1) The obtained strain responses are used to determine the candidate material plane of the dangerous position based on the coordinate transformation. Then, the critical damage plane is determined by means of the average weighted method.
(2) The cyclic rain flow counting method is applied to count the normal and shear strain response time histories on the critical plane. Then, the stress or strain components required in the SW model and the modified FS model are determined, and the load spectra of variable amplitude fatigue are compiled.
(3) The damage parameters and fatigue properties obtained from ELCF tests are substituted into the SW model and modified FS model, respectively. The fatigue life corresponding to each strain amplitude is calculated according to the load spectrum of variable amplitude fatigue.
(4) The fatigue damage is evaluated based on the Miner linear damage accumulation criterion, and the applicability of the two damage evaluation models is analyzed by comparing the fatigue damage assessment with the test results.

### 3.2. Determination of Critical Plane and Cyclic Rain Flow Counting

The critical plane approach has been widely accepted in multiaxial fatigue analysis. It is the primary method for performing strain state decomposition according to six strain components in which the strain tensors on the candidate material plane are calculated, and the location of the fatigue damage critical plane by means of the weighted average method is then determined.

The z-axis is chosen parallel to the loading axis at the beam end, and the x-axis coincides with the outside normal of the surface. Then, the strain tensor at the weld root can be expressed as

(3) $$\varepsilon =\left[\begin{array}{ccc} \varepsilon_{xx} & \varepsilon_{xy} & \varepsilon_{xz}\\ \varepsilon_{xy} & \varepsilon_{yy} & \varepsilon_{yz}\\ \varepsilon_{xz} & \varepsilon_{yz} & \varepsilon_{zz} \end{array}\right]$$

**Figure 10:**
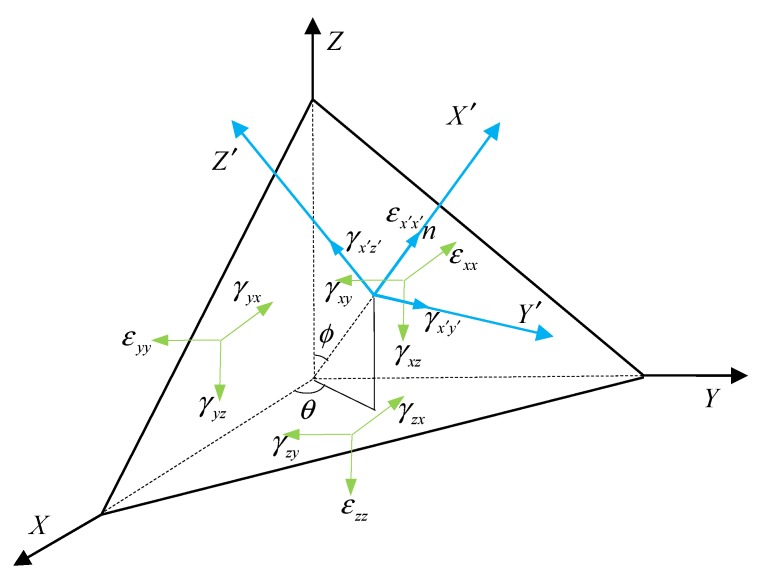
The strains on the candidate critical plane in a three-dimensional coordinate system.

A candidate material plane is characterized by the critical location with the orientation of the unit normal n to the plane defined by angles θ and ϕ [33], as shown in Figure 10. The strain tensors on the candidate material plane are calculated by computing the transformation matrices for a given set of θ and ϕ angles.

(4) $$\varepsilon^{\prime }=M^{T}\varepsilon M$$

where $\varepsilon^{\prime }$ is the strain tensor on the candidate material plane and $M^{T}$ is the transpose of the transformation matrices M; M is given by

(5) $$Μ=\left[\begin{array}{ccc} a_{11} & a_{12} & a_{13}\\ a_{21} & a_{22} & a_{23}\\ a_{31} & a_{32} & a_{33} \end{array}\right]=\left[\begin{array}{ccc} \cos\theta \sin\phi & \sin\theta \sin\phi & \cos\phi \\ -\sin\theta & \cos\theta & 0\\ -\cos\theta \cos\phi & -\sin\theta \cos\phi & \sin\phi \end{array}\right]$$

The shear strain component and normal strain component on the candidate material plane can be derived from Equations (4) and (5):

(6) $$\{\begin{array}{l} \begin{array}{c} \varepsilon_{x^{\prime }x^{\prime }}=0.25\left(1-\cos2\phi \right)\left[\varepsilon_{xx}\left(1+\cos2\theta \right)+\varepsilon_{yy}\left(1-\cos2\theta \right)+\gamma_{xy}\sin2\theta \right]\\ +0.5\left[\varepsilon_{zz}\left(1+\cos2\phi \right)+\left(\gamma_{xz}\cos\theta +\gamma_{yz}\sin\theta \right)\sin2\phi \right] \end{array}\\ \begin{array}{c} \gamma_{x^{\prime }y^{\prime }}=0.5\sin\phi \left(-\varepsilon_{xx}\sin2\theta +\varepsilon_{yy}\sin2\theta +2\gamma_{xy}\cos2\theta \right)\\ +\cos\phi \left(\varepsilon_{zz}\sin\phi +\gamma_{yz}\cos\theta -\gamma_{zx}\sin\theta \right) \end{array}\\ \begin{array}{c} \gamma_{x^{\prime }z^{\prime }}=0.25\sin2\phi \left[-\varepsilon_{xx}\left(1+\cos2\theta \right)+\varepsilon_{yy}\left(\cos2\theta -1\right)+2\varepsilon_{zz}-2\gamma_{xy}\sin2\theta \right]\\ -\cos2\phi \left(\gamma_{yz}\sin\theta +\gamma_{zx}\cos\theta \right) \end{array} \end{array}$$

**Figure 11:**
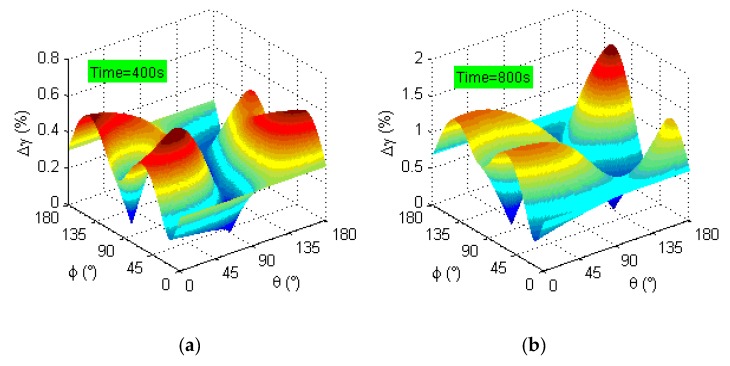
The location of the critical plane: (**a**) time = 400 s; (**b**) time = 800 s.

The shear and normal strains on the candidate material plane (θ,ϕ) can be expressed as a function of time t as Equation (6). Then, the shear strain range acting on the candidate material plane in the current cycle can be calculated to determine the maximum shear strain range $\Delta \gamma_{\max}$ and the location of the $\Delta \gamma_{\max}$ plane. For the weld root of NWB_2, the distribution curve surfaces of the critical plane at different times are shown in Figure 11.

It can be seen from Figure 11 that the maximum shear strain range $\Delta \gamma_{\max}$ on the same candidate material plane changes significantly with time at 400 and 800 s. That is to say, the location of the $\Delta \gamma_{\max}$ plane of the fatigue failure dangerous position at the weld zone changes continuously when the beam ends are controlled by reverse cyclic loading. It is well known that normal strain and shear strain are controlled in the same phase under multiaxial proportional loading, and that the strain principal axis only changes in size but not in direction. Thus, the location of the $\Delta \gamma_{\max}$ plane remains unchanged at any time. However, there is a phase difference between the normal strain and the shear strain under multiaxial nonproportional loading, i.e., the strain principal axis changes not only in size but also in direction. Therefore, a new $\Delta \gamma_{\max}$ plane may be generated, corresponding to a new different strain state under multiaxial nonproportional loading. That is why the location of $\Delta \gamma_{\max}$ plane is different at any time.

In terms of fatigue damage assessment, it is necessary that the critical damage plane is representative. It is considered that the normal and shear strain on the material plane often have several different peaks during the response time history; the angle of the critical damage plane $\bar{\theta }$ is determined by the weighted average method in the present study. $\bar{\theta }$ is given by

(7) $$\bar{\theta }=\frac{1}{W}\sum_{i=1}^{n}\theta \left(t_{i}\right)w\left(t_{i}\right)$$

where W is the sum of weights $w\left(t_{i}\right)$ and $\theta \left(t_{i}\right)$ is the maximum angle of the candidate material plane in the current time. $w\left(t_{i}\right)$ is the weight function of $\theta \left(t_{i}\right)$, which indicates that the maximum shear strain is related to the damage of the material; $w\left(t_{i}\right)$ is given by

(8) $$w\left(t_{i}\right)=\frac{\gamma_{t,\max}-\gamma_{\min}}{\gamma_{\max}-\gamma_{\min}}$$

where $\gamma_{\max}$, $\gamma_{\min}$ is the maximum and minimum shear strain in the whole strain time history, respectively. $\gamma_{t,\max}$ is the maximum shear strain at time t.

**Figure 12:**
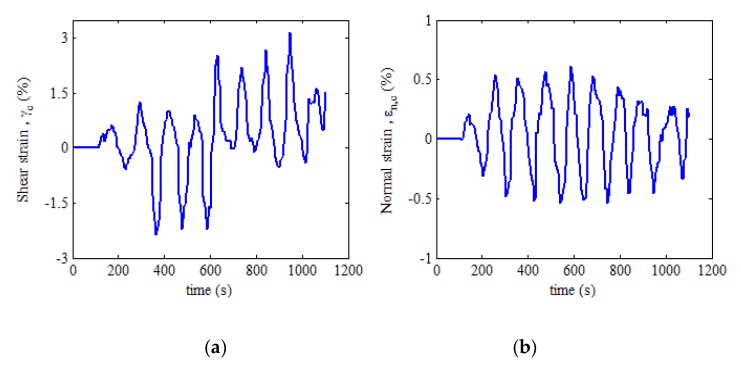
Strain response on the critical damage plane: (**a**) shear strain; (**b**) normal strain.

In light of the above steps, the location of the critical damage plane of the dangerous position at the weld zone of NWB_2 is calculated as $\theta =86^{\circ }$ and $\phi =73^{\circ }$ using MATLAB. The obtained critical plane angles are substituted into Equation (6). The maximum shear strain response on the critical plane $\gamma_{c}$ and the normal strain response on the maximum shear plane $\varepsilon_{n,c}$ are then determined, as shown in Figure 12.

**Figure 13:**
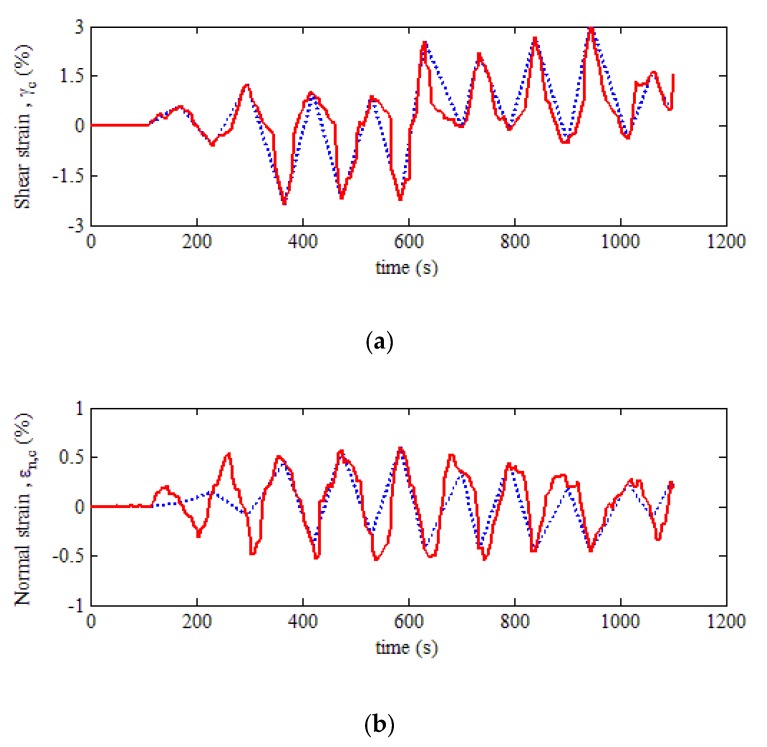
Double cyclic rain flow counting: (**a**) shear strain range; (**b**) normal strain range. (Red line represents strain responses; blue line represents strain range correlated to double rain flow counting method).

Based on the obtained strain responses on the critical damage plane, the double rain flow counting method can be used to compile the load spectrums of variable amplitude fatigue. In practice, the number of cycles, shear strain range Δγ, and the shear mean strain $\gamma_{m}$ are counted first, and the turning point of each cycle is also determined. The peaks of the dotted line are the turning points as shown in Figure 13a. Then, the maximum normal strain range Δε and the normal mean strain $\varepsilon_{m}$ between adjacent turning points are calculated from the normal strain response on the critical plane, as shown in Figure 13b.

The double cyclic rain flow counting results for the critical damage plane at the weld zone of NWB_2 are shown in Table 5.

**Figure 14:**
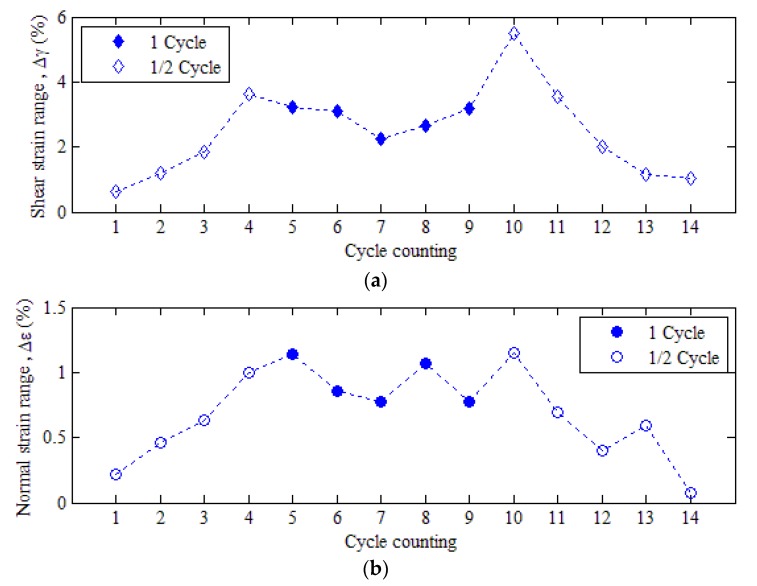
The load spectra of variable amplitude fatigue: (**a**) shear strain range; (**b**) normal strain range.

As shown in Table 5, the value of 1 presented in the number of cycles means one cycle, and 0.5 means half a cycle. In terms of the extracted loading process of NWB_2, the number of rain flow counts is 14, and the total number of cycles is 10.5 weeks. The load spectra of variable amplitude fatigue obtained from the statistical cyclic counting are plotted in Figure 14.

### 3.3. Multiaxial Fatigue Damage Parameters

In light of the above studies, the fatigue damage parameters for fatigue life prediction are determined by the results of the cyclic rain flow counting. The damage parameters of the SW model and modified FS model are as follows:

#### 3.3.1. Parameters of the SW model

It is indicated by the tension–torsion multiaxial fatigue test that the amplitude of $\varepsilon_{n}$ on the maximum shear plane is very small under proportional loading, whereas the amplitude of $\varepsilon_{n}$ obviously increases with the phase angle under nonproportional loading [36]. Furthermore, Shang and Wang point out that fatigue crack growth is a decohesion process along the crack tip shear band from the point of view of the micrometer, and the normal strain on the crack plane accelerates this behavior. This means that $\gamma_{\max}$ and $\varepsilon_{n}$ on the critical plane are two important fatigue damage-controlled parameters, and the size of the normal strain excursion between adjacent turning points is one of the parameters affecting fatigue crack growth. Based on the above ideas, Shang and Wang suggest that an equivalent strain to the von Mises criterion is used as a multiaxial fatigue damage parameter:

(9) $$\frac{\Delta \varepsilon_{eq}^{cr}}{2}={\left[\varepsilon_{n}^{*2}+\frac{1}{3}{\left(\frac{\Delta \gamma_{\max}}{2}\right)}^{2}\right]}^{\frac{1}{2}}$$

where $\Delta \varepsilon_{eq}^{cr}/2$ is the equivalent strain, $\varepsilon_{n}^{*}$ is the normal strain excursion between adjacent turning points, and $\Delta \gamma_{\max}$ is the maximum shear strain range on the critical plane.

The multiaxial fatigue damage formula relating to the Manson–Coffin equation is expressed as Equation (10), that is, the SW model:

(10) $$\frac{\Delta \varepsilon_{eq}^{cr}}{2}=\frac{{\sigma^{\prime }}_{f}}{E}{\left(2N_{f}\right)}^{b}+{\varepsilon^{\prime }}_{f}{\left(2N_{f}\right)}^{c}$$

In the case of proportional loading, Equation (10) reduces the equivalent strain approach form. In the case of uniaxial loading, Equation (10) reduces the Manson–Coffin equation to a uniaxial form. Thus, Shang and Wang proposed that Equation (10) could be used as a unified fatigue damage criterion under either proportional loading (including uniaxial and multiaxial loading) or nonproportional loading.

#### 3.3.2. Parameters of the Modified FS model

In terms of multiaxial fatigue study, researchers have made great progress in the area of damage accumulation and life prediction based on the strain components or the combined strain with stress components on the critical plane. The other popular approach is that proposed by Fatemi and Socie. They introduced a maximum normal stress $\sigma_{n,\max}$ on the maximum shear strain plane to reflect the effect of additional cyclic hardening under nonproportional loading [32]. The FS damage parameter is expressed as

(11) $$\frac{\Delta \gamma_{\max}}{2}\left(1+k\frac{\sigma_{n,\max}}{\sigma_{y}}\right)=\left[\left(1+\upsilon_{e}\right)\frac{{\sigma^{\prime }}_{f}}{E}{\left(2N_{f}\right)}^{b}+\left(1+\upsilon_{p}\right){\varepsilon^{\prime }}_{f}{\left(2N_{f}\right)}^{c}\right]\left(1+k\frac{{\sigma^{\prime }}_{f}}{2\sigma_{y}}{\left(2N_{f}\right)}^{b}\right)$$

where k is an empirical constant, $\sigma_{y}$ is the yield strength, and $\upsilon_{e}$, $\upsilon_{p}$ are elastic Poisson ratio and plastic Poisson ratio, respectively. The other symbols are as previously stated. It is worth noting here that the stress-correlated factor, $\frac{1}{2}\left(1+k\frac{\sigma_{n,\max}}{\sigma_{y}}\right)$, found in Equation (11), is not constant. The maximum normal stress $\sigma_{n,\max}$ is determined by the modified Ramberg–Osgood relation [31]:

(12) $$\varepsilon_{n,\max}=\frac{\sigma_{n,\max}}{E}+{\left[\frac{\sigma_{n,\max}}{K^{\prime }\left(1+L\cdot \Phi \right)}\right]}^{\frac{1}{n^{\prime }}}$$

where $\varepsilon_{n,\max}$ is the maximum normal strain, $K^{\prime }$ and $n^{\prime }$ are as previously stated, and L is the nonproportional additional hardening coefficient, which depends on the material. L is determined by [37]

(13) $$L=\frac{{\bar{\sigma }}_{\mathrm{OP}}}{{\bar{\sigma }}_{\mathrm{IP}}}-1$$

where ${\bar{\sigma }}_{\mathrm{OP}}$, ${\bar{\sigma }}_{\mathrm{IP}}$ are the equivalent stress amplitude under 90° out-of-phase loading and the equivalent stress amplitude under proportional loading, respectively. Φ is the nonproportionality of the strain path determined by [31]

(14) $$\Phi =2\frac{A_{\theta ,\max}}{A_{\max}}-1$$

where $A_{\theta ,\max}$ is the swept area of the $\Delta \gamma_{\theta ,\max}-\theta$ polar coordinate space and $A_{\max}$ is the circle area with a radius of the maximum shear strain during one cycle.

It is believed that Equation (12) can account for the nonproportionality of both material additional hardening and the strain path. However, it is worth noting that not all metallic materials exhibit nonproportional additional hardening characteristics under nonproportional loading. The value of L is zero for materials without a nonproportional additional hardening effect, and the value of the modified coefficient (1+L⋅Φ) in Equation (12) is then zero, even under nonproportional loading. This means that Equation (12) is similar to the Ramberg–Osgood relation, i.e., Equation (2). That is to say, Equation (12) cannot accurately reflect the effect of additional hardening when applied to fatigue life prediction for a material without a nonproportional additional hardening effect. To solve this problem, a new modified coefficient $k^{*}$ is introduced in Equation (11) to reflect the effect of additional hardening by our research team. In addition, it is suggested that $\sigma_{n,\max}$ can be replaced by ${\bar{\sigma }}_{n,\max}$. Therefore, our previously proposed modified FS model is expressed as

(15) $$\frac{\Delta \gamma_{\max}}{2}\left(1+k^{*}\frac{{\bar{\sigma }}_{n,\max}}{\sigma_{y}}\right)=\left[\left(1+\upsilon_{e}\right)\frac{{\sigma^{\prime }}_{f}}{E}{\left(2N_{f}\right)}^{b}+\left(1+\upsilon_{p}\right){\varepsilon^{\prime }}_{f}{\left(2N_{f}\right)}^{c}\right]\left(1+k^{*}\frac{{\sigma^{\prime }}_{f}}{2\sigma_{y}}{\left(2N_{f}\right)}^{b}\right)$$

where ${\bar{\sigma }}_{n,\max}$ is the maximum normal stress on the critical plane determined by the Ramberg–Osgood relation. The other symbols are as previously stated except $k^{*}$, which is defined as the nonproportional factor, and is given by [35]

(16) $$k^{*}=1+\sqrt{\Phi^{2}+{\left(L\cdot \Phi \right)}^{2}}=1+\Phi \sqrt{1+L^{2}}$$

It can be observed from Equation (16) that the value of L is zero for materials without a nonproportional additional hardening effect, whereas the effect of nonproportionality of the strain path is not ignored. That is to say, $k^{*}$ can account for the effect of the nonproportionality of both the strain path and material additional hardening. In other words, the proposed modified FS model for life prediction appears to be relatively accurate in adopting the modified coefficient $k^{*}$, and may be used for different materials.

**Figure 15:**
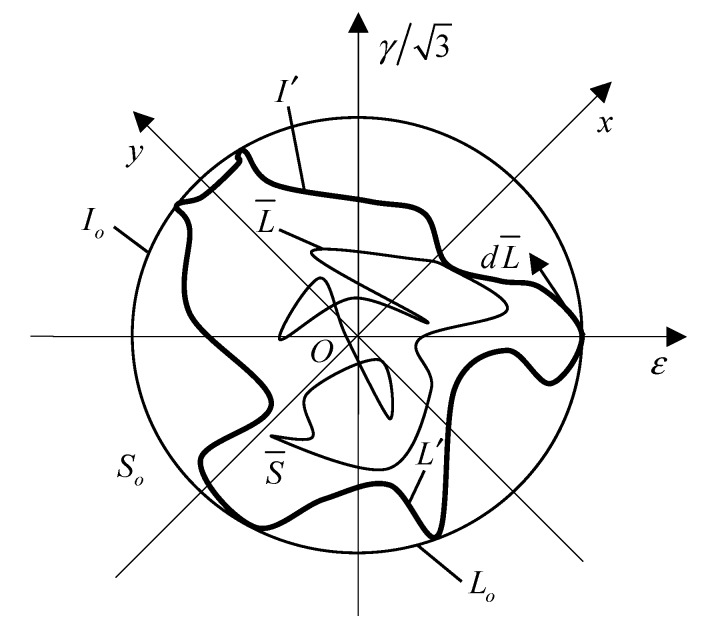
Random strain path and its envelope path.

The nonproportionality of the strain path cannot be adequately accounted for if determined by Equation (14). This is because the value of Φ may be negative or zero when $A_{\theta ,\max}$ is less than or equal to $A_{\max}/2$, resulting in limitations of application. To solve this problem, we carry out a second amendment for the nonproportional factor $k^{*}$. Φ is determined by the introduction of the moment of inertia [34]. As shown in Figure 15, $\bar{L}$ is the random strain path simplified by its enveloping line, i.e., the equivalent convex path $L^{\prime }$, and the maximum chord length of $\bar{L}$ is the diameter of the maximum circle path $L_{o}$. It is worth noting that the midpoint of the maximum chord length is used as the origin of the $\varepsilon -\gamma /\sqrt{3}$ coordinate system, the x-axis represents the in-phase proportional loading path, and the y-axis represents the antiphase proportional loading path.

Φ is calculated using the following formula:

(17) $$\Phi ={\left(I^{\prime }/I_{o}\right)}^{h}$$

where $I^{\prime }$, $I_{o}$, and h are respectively given by

(18) $$I^{\prime }=\int_{S^{\prime }}y^{2}dS^{\prime }$$

(19) $$I_{o}=\int_{S_{o}}y^{2}dS_{o}$$

(20) $$h=\left(1-\frac{\bar{S}}{S_{o}}\right)\frac{L_{cyc}}{4\Delta r_{\max}}$$

where $I^{\prime }$ is the moment of inertia of $L^{\prime }$ to the x-axis in Equation (18), which indicates the deviation of the point from the in-phase proportional loading path. In other words, the moment of inertia can account for the nonproportional contribution of the strain path. Any point in area $S^{\prime }$ surrounded by $L^{\prime }$ contributes to nonproportional additional hardening, and y represents the distance from any point in area $S^{\prime }$ to the x-axis. Similar to $I^{\prime }$, $I_{o}$ is the moment of inertia of $L_{o}$ to the x-axis. $S_{o}$ is the circle area with diameter of the maximum chord length. y is used to express the distance from any point in area $S_{o}$ to the x-axis in Equation (19). Because an equivalent convex path may appear even under different random strain path loading, the contribution of the random strain path is taken into account by the parameter h. $\bar{S}$ is the area surrounded by the random strain path $\bar{L}$. $L_{cyc}$ and $\Delta r_{\max}$ are the perimeter and the maximum strain range of $\bar{L}$, respectively.

**Figure 16:**
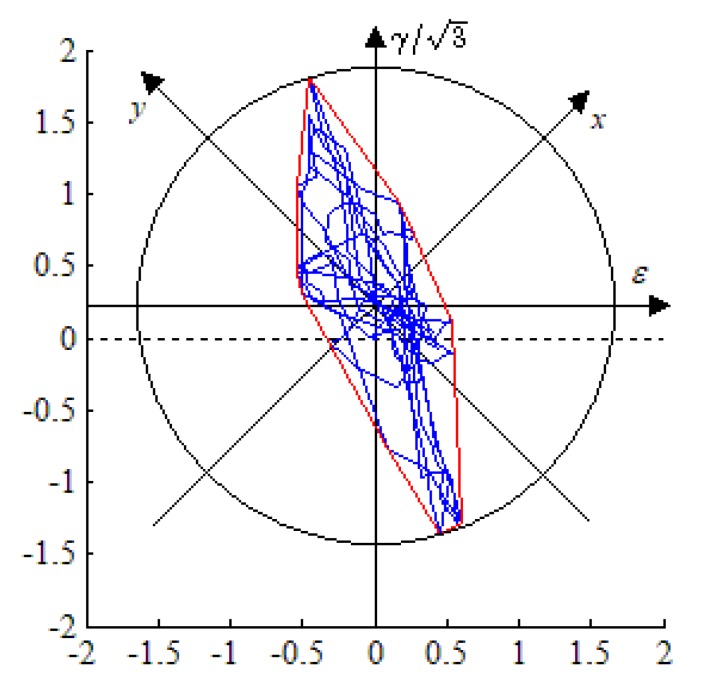
Strain path and equivalent convex path on the critical plane of NWB_2.

The strain path of the dangerous position at the weld zone of NWB_2 is shown in Figure 16. The dotted line represents the original coordinate position and the new coordinate position is determined by the midpoint of the maximum chord length of the strain path marked with the blue line. Its equivalent convex path and maximum circular path are marked with the red and black lines, respectively.

It can be seen from Figure 16 that the strain path changes constantly and has obvious randomness, while the determination of Φ is convenient for engineering applications when the convex hull in the MATLAB program is adopted, and its physical significance is clear.

In light of the above study, the nonproportional parameters in the modified FS model are shown in Table 6.

### 3.4. Damage Accumulation Approach

Fatigue damage is an irreversible process in which material properties deteriorate continuously under reverse cyclic loading. It is also a process in which damage accumulates gradually. Generally speaking, most of the components are subjected to variable amplitude cyclic loading. Therefore, it is very important for fatigue damage evaluations to study the damage accumulation rule and fatigue failure criteria under variable amplitude cyclic loading.

It is known that the Miner linear damage accumulation rule can predict the mean life of engineering structures under random loading. The fatigue damage per cycle, $D=1/N_{f}$, can be determined from the fatigue criteria for each parameter. Therefore, the Miner linear damage accumulation rule is used to calculate the cumulative fatigue damage in this paper, and the damage value D=1 expresses fatigue crack initiation.

## 4. Discussion

The obtained fatigue damage parameters are respectively substituted into Equations (10) and (15) to calculate the fatigue lives $N_{i}$ for each strain amplitude. Then, the fatigue damage per cycle is determined. The calculated results of cumulative damage ranging from the 3rd to the 8th cycle are shown in Table 7.

As shown in Table 7, it can be seen that:

(1) In terms of the same fatigue properties of either CLG or PB, ranging from the 3rd to the 8th cycle, the damage values of the SW model are much smaller than those of the modified FS model. From the comparison between the experiment and damage evaluation, it is indicated that for box-column joints, the SW model is inclined to underestimate the risk. This may be because the nonproportionality of material additional hardening and the strain path both aggravate fatigue damage under nonproportional loading [31], whereas the nonproportional factor is not taken into account in the SW model.
(2) In terms of the same fatigue damage model, either SW or the modified FS model, the fatigue values calculated by the fatigue properties of CLG are much larger than those calculated by the fatigue properties of PB, which are more coincident with the reverse cyclic loading test results. This may be because the geometric shape and force characteristics of the CLG specimens are more similar to those of box-column joints than those of PB specimens. It is shown that the structural details, i.e., geometric shape and force characteristics, play an important role in damage evaluation; the more similar the structural details, the better the damage estimation.
(3) In terms of the modified FS model using the fatigue properties of CLG, the calculated damage value of the dangerous position at the weld zone of NWB_2 is 1.0321 at the 7th cycle, which is slightly larger than *D* = 1. The Miner linear damage accumulation rule indicates that a microscopic crack will be initiated at the time when the 7th cycle is coming to an end, while it was previously stated in Section 2.2 that tiny visible crack initiation could be observed just after the 7th cycle, that is, at the time period between the 7th and 8th cycle. However, there is no contradiction between the theoretical calculation results and the experimental results. There are two reasons for this. The first is that from the point of view of a micrometer, fatigue crack is a process ranging from microcosmic to visible, which takes some time. Furthermore, the observation of a crack is limited by the test instruments. The second reason is that the error between the theoretical calculation results and the experimental results is very small, which is acceptable. Therefore, it is shown that the damage assessment from adopting this model is relatively accurate.

In summary, for ELCF damage evaluations of box-column welded joints, the method can provide good estimations in cases where the fatigue properties of CLG are adopted as the proposed modified FS model parameters.

## 5. Conclusions

Based on numerous existing studies, it is difficult to accurately assess the damage of a steel structure during the ELCF fracture process using the macroparameters of the whole structure or a structure component, since local structural details and fatigue crack initiation mechanics are ignored. A damage evaluation methodology based on the structural details of I-beam to box-column welded joints, extended from multiaxial fatigue critical plane theory, is proposed to assess ELCF crack initiation life and damage. Based on experimental studies associated with analytical studies, the key conclusions are as follows:

(1) The failure of beam-to-column welded joints is multiaxial and nonproportional ELCF when subjected to severe seismic excitation.
(2) From a comparison between the FS model containing a nonproportional factor and the SW model without this factor, it was shown that the effect of nonproportional additional hardening plays a key role in damage evaluation.
(3) The nonproportional factor in the FS model previously proposed by us was again modified and introduced to reflect the effect of nonproportionality. This accounts for both the strain path and additional material hardening. It was found that the fatigue damage model including this proposed factor can successfully evaluate the fatigue damage of beam-to-column welded joints with reasonable accuracy.
(4) The fatigue properties of PB and CLG when considering welded structural details were respectively adopted as the damage parameters in the modified FS model and the SW model. From the comparison between experiments and damage estimation, it was shown that the fatigue properties of CLG result in the damage evaluation being more consistent with the test results than that of PB because of the highly similar geometric shape and force characteristics.

In conclusion, the modified FS model with the fatigue properties of CLG is most in line with the test results, which verifies the applicability of the model. The presented work makes an important contribution to ELCF damage evaluations of steel frameworks.

## Figures and Tables

**Table 1:** Relevant mechanical parameters.

Young’s Modulus E(GPa)	Yield Strength $\sigma_{y}\left(\mathrm{MPa}\right)$	Ultimate Strength $\sigma_{u}\left(\mathrm{MPa}\right)$	Ultimate Strain $\varepsilon_{u}\left(\%\right)$	Elastic Poisson Ratio $\upsilon_{e}$	Plastic Poisson Ratio $\upsilon_{p}$
200	254.522	415.449	20.976	0.3	0.5

**Table 2:** Strain amplitude used in the tests and test results.

Specimen No.	$\frac{\Delta \varepsilon }{2}$	$\frac{\Delta \varepsilon_{p}}{2}$	$\frac{\Delta \varepsilon_{e}}{2}$	$\begin{array}{l} \Delta \sigma /2\\ \left(\mathrm{MPa}\right) \end{array}$	$2N_{f}$	Specimen No.	$\frac{\Delta \varepsilon }{2}$	$\frac{\Delta \varepsilon_{p}}{2}$	$\frac{\Delta \varepsilon_{e}}{2}$	$\begin{array}{l} \Delta \sigma /2\\ \left(\mathrm{MPa}\right) \end{array}$	$2N_{f}$
PB04	0.004	0.0024	0.0016	319	464	CLG04	0.004	0.0024	0.0016	316	810
PB05	0.005	0.0032	0.0018	355	210	CLG05	0.005	0.0033	0.0017	329	460
PB06	0.006	0.0042	0.0018	356	140	CLG06	0.006	0.0043	0.0017	353	164
PB07	0.007	0.0050	0.0020	364	98	CLG07	0.007	0.0052	0.0018	403	84
PB08	0.008	0.0060	0.0020	395	80	CLG08	0.008	0.0060	0.0020	389	66
PB10	0.010	0.0078	0.0022	415	62	CLG10	0.010	0.0079	0.0021	419	30

**Table 3:** Fatigue and cyclic stress–strain properties of welded structural details.

Specimen	$K^{\prime }(\mathrm{MPa})$	$n^{\prime }$	${\sigma^{\prime }}_{f}(\mathrm{MPa})$	b	${\varepsilon^{\prime }}_{f}$	c
PB	1134	0.2085	783.3	−0.1397	0.1193	−0.6739
CLG	1369	0.2430	576.24	−0.0830	0.0273	−0.3611

**Table 4:** Loading condition.

Specimen No.	Displacement of Beam Ends (ΔU/mm)
1	± 15 → ± 17 → ± 20 → ± 21 → ± 22 → ± 23 → ± 25 → ± 27
2	± 17
3	± 14 → ± 16 → ± 18 → ± 20

**Table 5:** Double cyclic rain flow counting results of the maximum damage critical plane of NWB_2.

No.	Number of Cycles	$\gamma_{m}\left(\%\right)$	Δγ(%)	$\varepsilon_{m}\left(\%\right)$	Δε(%)
1	0.5	0.311026	0.622408	0.102318	0.217569
2	0.5	0.0149855	1.21449	−0.07448	0.465991
3	0.5	0.32991	1.84434	0.220915	0.636
4	0.5	−0.5557	3.61556	0.01509	0.997263
5	1	−0.60909	3.23508	0.03512	1.144744
6	1	−0.662328	3.10254	0.141659	0.855743
7	1	1.09227	2.24082	−0.03551	0.771647
8	1	1.20526	2.64228	−0.00706	1.072557
9	1	1.07013	3.19876	−0.06797	0.775517
10	0.5	0.390535	5.50803	0.032078	1.150827
11	0.5	1.37465	3.5398	−0.10587	0.693131
12	0.5	0.617375	2.02525	0.076209	0.400422
13	0.5	1.05633	1.14734	−0.03933	0.594464
14	0.5	1.00771	1.0501	0.221689	0.072419

**Table 6:** Nonproportional parameters in the modified FS model.

Nonproportionality of Strain Path Φ	Nonproportional Additional Hardening Coefficient L	Nonproportional Factor $k^{*}$
0.4425	0.31	1.4425

**Table 7:** Comparison of fatigue damage models using fatigue properties of structural details for NWB_2.

Fatigue Damage Model	Fatigue Properties	Cumulative Damage					
		3rd	4th	5th	6th	7th	8th
**SW model**	CLG	0.1464	0.2089	0.2327	0.2827	0.3452	0.5535
	PB	0.0751	0.1096	0.1300	0.1603	0.1948	0.2564
**Modified FS model**	CLG	0.4059	0.6059	0.6892	0.8321	1.0321	1.3821
	PB	0.1658	0.2427	0.2903	0.3570	0.4339	0.5794
